# Compensation for Lateral Misalignment in Litz Wire Based on Multilayer Coil Technology

**DOI:** 10.3390/s21072295

**Published:** 2021-03-25

**Authors:** Hyungjun Chang, Taejun Lim, Yongshik Lee

**Affiliations:** Department of Electrical and Electronic Engineering, Yonsei University, Seoul 03722, Korea; hyungjunchang@yonsei.ac.kr (H.C.); taejunim@yonsei.ac.kr (T.L.)

**Keywords:** wireless power transfer, multilayer coils, transfer efficiency, lateral misalignment

## Abstract

This study applies a multilayer coil technology that can compensate for a decrease in transfer efficiency due to a lateral misalignment in a practical 100 kHz-band wireless power transfer system and validates its effect on the efficiency of compensation. The effectiveness is investigated using coils fabricated with Litz wires. Three-turn rectangular assistant coils 22.4 × 45.3 mm^2^ in size were stacked on a five-turn circular primary coil with a diameter of 45.3 mm in a 2 × 1 array. Transfer efficiency between two such coils was measured by producing lateral misalignment, while maintaining the vertical distance between the Tx and Rx coils at 7 mm. The experimental results showed that the transfer efficiency was compensated by approximately 46.1%P maximum in a misalignment state of 30 mm, which corresponded to 67% of the maximum size of the coil, compared to the transfer efficiency of the structure, in which the multilayer coil was not applied. Furthermore, transfer efficiency was compensated by 37.6%P, even in an asymmetric system in which the multilayer structure was applied only to the Tx coil, thereby confirming an excellent multilayer coil technology effect on compensation for lateral misalignment in practical cases.

## 1. Introduction

Wireless charging based on wireless power transfer technology is more convenient than wired charging, and has quickly gained sufficient popularity to be included as a basic feature in current mobile devices. Performance has advanced to demonstrate a charging speed comparable to wired charging; its application range is expected to include medium and large devices, such as tablets, laptop computers, and automobiles, in the future.

However, when a misalignment occurs between the transmit and receive (Tx/Rx) coils, the wireless power transfer efficiency drops rapidly. This problem remains to be solved effectively. Recently, various techniques have been reported to extend the effective range of wireless power transfer. For instance, distance-adaptive wireless power transfer systems have been demonstrated based on novel adaptive matching techniques [1,2,3], by over-coupling the coils [4], or by utilizing compensating capacitors [5]. Although these methods have demonstrated their potential with an efficiency of as high as 80% for a distance of 50% (relative to the largest coil dimension) between the Tx and Rx coils, the effect is limited to cases where the two coils are well, if not perfectly, aligned. In [6], a transfer efficiency of 80% at a misalignment of 67% relative to the maximum coil dimension was achieved by maximizing the fringing field. However, it requires relatively large coils, which may not be compatible at kHz frequencies. Otherwise, they have complex structures, are impractical [7,8,9,10,11], or the maximum degree of misalignment to compensate for the decreased efficiency is limited [12].

The technique [13,14] first proposed to compensate for the reduced efficiency was changing the size of coils, but it had low practicality because the coil size must be changed according to the degree of misalignment. As a realistic implementation strategy of this approach, a multilayer coil technology [15] is proposed, which compensates this efficiency drop. Several coils of different sizes are stacked to form a single coil stack, and the Tx/Rx coil pair with the highest transfer efficiency according to the misalignment, i.e., the most suitable alignment state, is selected. However, the compensation effect has been verified only for rectangular coils, particularly at 6.78 MHz for planar coils printed with copper on dielectrics.

This study applies the multilayer coil technology of [15] to circular coils consisting of non-planar Litz wires, and measures their transfer efficiency around 100 kHz, to experimentally investigate the misalignment compensation effect in more practical systems with respect to the current wireless charging market.

## 2. Materials and Methods

### 2.1. Multilayer Coil Technology

**Figure 1:**
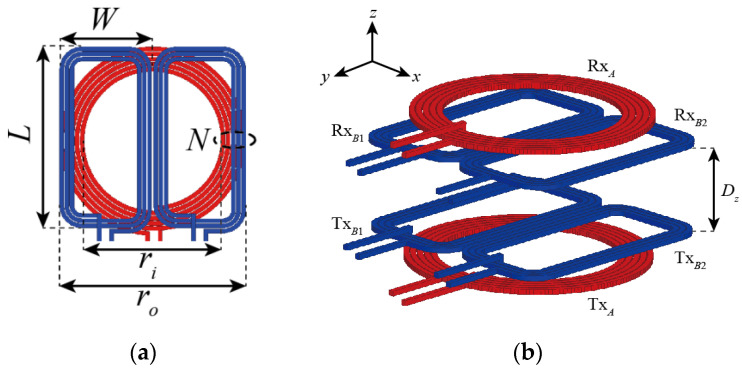
Configuration of: (**a**) 2 × 1 multilayer coil; (**b**) symmetric wireless power transfer (WPT) coil system based on proposed multilayer coils.

Figure 1 shows the components of the multilayer coil technology. Each Tx/Rx coil stack is configured with an *m × n* assistant coil array added onto the primary coil. Here, *m* must be at least 2 to compensate for misalignments in the *x*-axis direction, and *n* must be at least 2 to compensate for low-dimensional lateral misalignment in the *y*-axis direction. Thus, the array size of the assistant coil must be at least 2 × 2 in order to compensate lateral misalignment in an arbitrary direction. In this study, it is assumed that misalignment occurs only in the *x*-axis direction, so the array size of the assistant coil is set to 2 × 1, as the investigation focuses on the compensation effect of the multilayer coil technology applied to the Litz wire coil. Figure 1 shows a 2 × 1 array of rectangular assistant coils added onto a circular primary coil, as an example.

**Figure 2:**
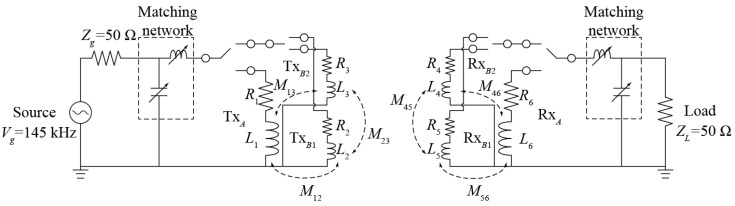
Equivalent circuit of a symmetric wireless power transfer (WPT) system based on 2 × 1 multilayer coils.

Figure 2 shows the equivalent circuit of a wireless power transfer system, based on the proposed multilayer coils in a 2 × 1 configuration. For simplicity, a symmetric system is assumed in which the Tx and Rx coils are identical.

**Figure 3:**
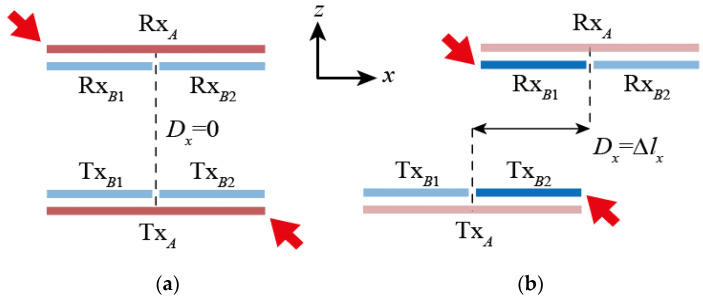
Operation of the multilayer coil system: (**a**) when well-aligned or with small misalignment; or (**b**) with large misalignment. Arrows indicate operating coils.

The basic principle of the stacked coil technique is depicted for an example 2 × 1 array assistant coil, as shown in Figure 3. The technique compensates for the lateral misalignment by operating the coils with the best alignment state, in a certain misalignment state, among the total of nine Tx/Rx coil pairs, which are possible combinations of the Tx/Rx coil stacks consisting of a total of three coils (one primary coil and two assistant coils). For example, when the Tx/Rx coil stack is properly aligned, as shown in Figure 3a, the alignment between the Tx*_A_* and Rx*_A_*, the primary coils of two coil stacks, is ideal; therefore, the power is transmitted by switching these coils. Naturally, in this case, the alignment is also good between an assistant coil pair Tx*_B_*_1_ and Rx*_B_*_1_, or Tx*_B_*_2_ and Rx*_B_*_2_, but because the assistant coil is less efficient than the primary coil due to its smaller size [15], it is best to switch Tx*_A_* and Rx*_A_*.

As shown in Figure 3b, if the misalignment occurs in the +*x* direction, and the alignment state between Tx*_B_*_2_ and R_X*B*1_ is better than between Tx*_A_* and Rx*_A_*, the efficiency drop due to the misalignment between the coil stacks can be compensated by switching these two coils. Furthermore, the misalignment occurring in the –*x*-axis direction can be compensated by switching the Tx*_B_*_1_ and Rx*_B_*_2_ coil pair, since the assistant coil array is 2 × 1. Much larger misalignment may occur, and the alignment state may be good for not only the Tx*_A_* and Rx*_A_* coil pair, but also the Tx*_Bi_* and Rx*_Bi_* (*i* = 1, 2) coil pair. In this case, it is generally most efficient to switch the Tx*_A_* and Rx*_A_* coil pair, which has the largest coil size.

### 2.2. Design and Simulation Test Results of the Multilayer Coil Stacks

Multilayer coil stacks based on a 2 × 1 array assistant coil were designed to verify the misalignment compensation effect of the proposed technology. The diameter of the circle was set to 45.3 mm according to the Qi A11 standard [16]. Since the array size of assistant coil is 2 × 1, the width of the assistant coil should be less than ^1^/_2_ of the primary coil width. The size of the assistant coil was 22.4 × 45.3 mm^2^ and the gap between the two assistant coils was 0.46 mm, which were arranged with a vertical gap of 1 mm from the primary coil. If this vertical gap is too small, it may suffer from excessive loss due to increased Eddy currents on secondary coils that are not operating, for instance, the two secondary coils when the Tx and Rx coils are perfectly aligned. If it is too large, the efficiency may decrease due to reduced coupling, and the volume will increase. All coils must be designed to maximize the *Q* since it is one of the most critical factors that determines the overall efficiency regardless of the misalignment. For example, the optimal number of turns for the primary coil and the secondary coil is 5 and 3, respectively. To avoid ripples in the transfer efficiency as the misalignment increases, the width of the secondary coil must be as large as possible, and the spacing between the two must be as small as possible. The final design parameters of the coils shown in Figure 1 are summarized in Table 1.

**Figure 4:**
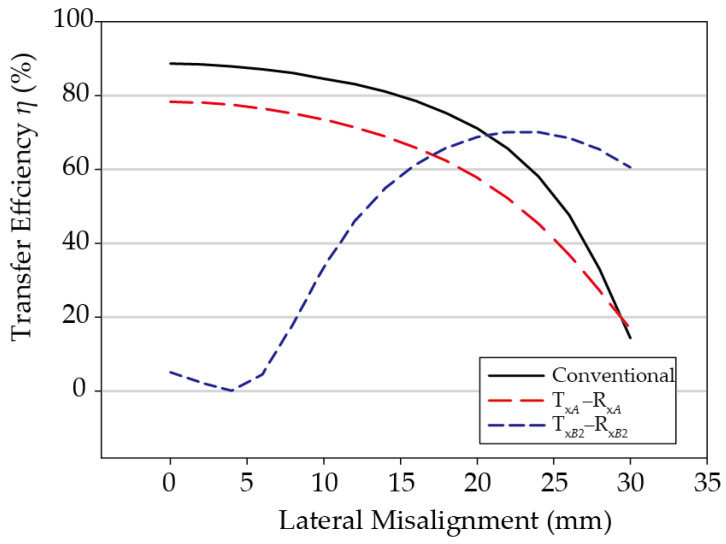
Simulated results of multilayer coils.

The transfer efficiency according to misalignment *D_x_* in the +*x*-axis direction between two identical coil stacks was calculated and is shown in Figure 4. Since the assistant coil array is 2 × 1 and the structure is perfectly symmetrical, the simulation test was performed only for the misalignment in the +*x*-axis direction. The frequency used was 145 kHz, and the maximum transducer gain G*_max_* was calculated through post-processing of the simulated efficiency using a high frequency structure simulator (HFSS). Here, *G_max_* is the maximum gain assuming that the input and output are simultaneously conjugate matched. This is a popular method of evaluating the performance of coils in WPT systems [17,18,19,20], which can be achieved with adaptive matching networks [1,2,3]. For comparison, the maximum transfer efficiency simulation results were also shown for a pair of conventional coils, which is the same as the primary coil of a multilayer coil stack, but to which the multilayer coil technology was not applied. All simulation results were obtained maintaining a minimum distance of 7 mm between the Tx and Rx coils.

As illustrated in Figure 4, the maximum transfer efficiency between the primary coils Tx*_A_* and Rx*_A_* of the proposed multilayer coil stack in the perfectly aligned state of *D_x_* = 0 mm is 78.3%, which is 10.3%P lower than when the multilayer coil is not applied. This is because the distance between the primary coils is 11.5 mm in the case of the multilayer coils, which is 4.5 mm more than the 7 mm gap when not using the multilayer coils, and additional loss occurs due to Eddy currents induced in the assistant coils located between the primary coils [21,22].

On the other hand, in the case of a pair of assistant coils in the *D_x_* = 0 mm state, the efficiency is low because the misalignment is large. As the misalignment *D_x_* increases, the alignment between these two assistant coils improves, and therefore the efficiency between the two assistant coils increases gradually. If the degree of misalignment increases to *D_x_* = 23 mm, Tx*_B_*_2_ and Rx*_B_*_1_ are perfectly aligned, and in this instance, the transfer efficiency is maximized to 70.2%.

Depending on the degree of lateral misalignment, the coil pairs with the largest coupling coefficient can be selected to maximize the transfer efficiency. For instance, when *D_x_* = 0 mm, the coupling coefficient, calculated using Ansys Maxwell simulator [23], between Tx*_A_* and Rx*_A_* is 0.262, which is substantially larger than the coupling coefficient of −0.024 between Tx*_B_*_2_ and Rx*_B_*_1_. The coupling coefficient between the former pair remains larger than that between the latter, until *D_x_* = 19 mm. Beyond this point, power should be transferred between the assistant coils since they are better aligned, and the coupling coefficient between them is higher than that between the primary coils. For example, when *D_x_* = 22 mm, the calculated coupling coefficients Tx*_B_*_2_ and Rx*_B_*_1_ is 0.26, much larger than the coupling coefficient between Tx*_A_* and Rx*_A_*, 0.094. Table 2 summarizes the calculated coupling coefficients for various *D_x_*. For comparison, those obtained by fitting the equivalent model to the full-wave simulated HFSS results are also provided, which are in excellent agreement with the those calculated by Maxwell.

In brief, when Tx*_A_* and Rx*_A_*, or Tx*_B_*_2_ and Rx*_B_*_1_, are switched according to the degree of misalignment in the +*x* direction, the efficiency graph by misalignment follows the envelope curve of the two efficiency graph lines in Figure 4, and the efficiency drop caused by misalignment can be significantly compensated. For example, the misalignment section having a transfer efficiency of over 65% can be increased from the conventional 22 mm to 28 mm, and the section having over 40% efficiency can be increased from 27 mm to 30 mm or more.

## 3. Results

**Figure 5:**
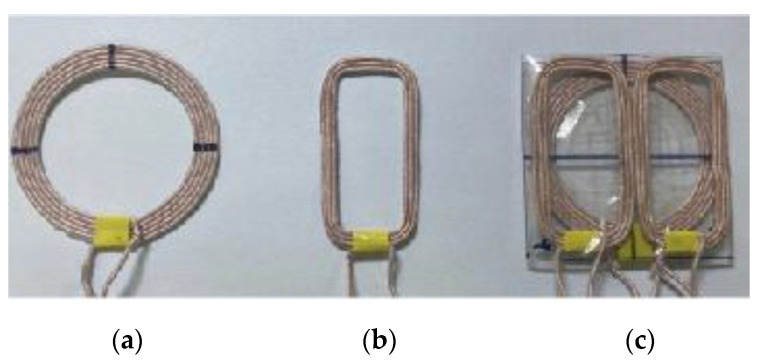
Photograph of the fabricated coils: (**a**) primary coil; (**b**) assistant coil; (**c**) multilayer coil.

The designed coils were fabricated by winding Litz wire. The Litz wire used was wire-wound type 17 American Wire Gauge (AWG) standard with a 1.15 mm diameter. Figure 5 shows images of the fabricated coils. For experimental verification of efficiency compensation according to misalignment, the efficiency was measured at 145 kHz using a Rohde & Schwarz ZNB8 vector network analyzer, and the maximum transducer gain G*_max_* was obtained through post-processing.

Table 3 compares the electrical parameters of the coils obtained from full-wave simulations and experiments. The discrepancy is due to the method of modeling the Litz wire in full-wave simulations, which is done in this work using a single wire with an effective conductivity instead of using multistrand wires. However, there is still a reasonable agreement between the two. The mutual inductances in Figure 2 are *M*_12_ = *M*_56_ = 0.27 μH, *M*_13_ = *M*_46_ = 0.27 μH, and *M*_23_ = *M*_45_ = 0.06 μH.

### 3.1. Symmetric System

**Figure 6:**
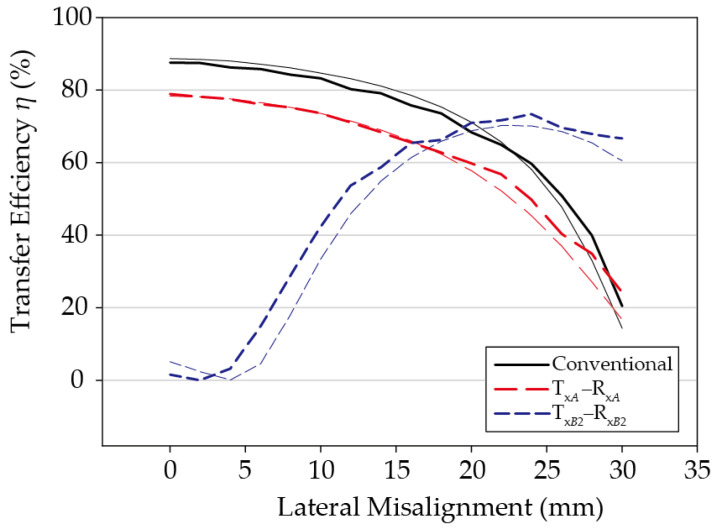
Comparison of the simulated (thin) and the measured (thick) transfer efficiencies between the two multilayer coils.

Measured results for the symmetric system in which the multilayer coil is applied to both Tx and Rx coils are shown in Figure 6. For comparison, experiments were conducted on the Tx/Rx coil system consisting of only a pair of primary coils, Tx*_A_* and Rx*_A_* to which the multilayer coil technology was not applied. All experiments were conducted while maintaining the minimum distance of 7 mm between coils, as in the simulation tests. Using an in-house measurement device, the lateral misalignment was measured up to *D_x_* = 30 mm by adjusting in 2 mm increments.

Since the Tx stack and the Rx stack are perfectly symmetrical, the efficiency was measured according to the degree of misalignment in the +*x* direction only. The measurement results were very similar to the simulation results; the efficiency when perfectly aligned was 87.5% in the case of not applying the multilayer coil, and 78.9% between Tx*_A_* and Rx*_A_* in the case of using the multilayer coil. In the case of applying the multilayer coil, there is still a phenomenon that the efficiency between the primary coils drops in the initial alignment state due to assistant coils. When the misalignment increases, the two coil systems all show decreased transfer efficiency. In the case of *D_x_* = 24 mm, the structure that does not use the multilayer coil shows a transfer efficiency of 59.6%. In comparison, the transfer efficiency is 73.4% between Tx*_B_*_2_ and Rx*_B_*_1_ in the case of using the multilayer coil, which indicates that the efficiency is compensated by about 13.8%P.

### 3.2. Asymmetric System

**Figure 7:**
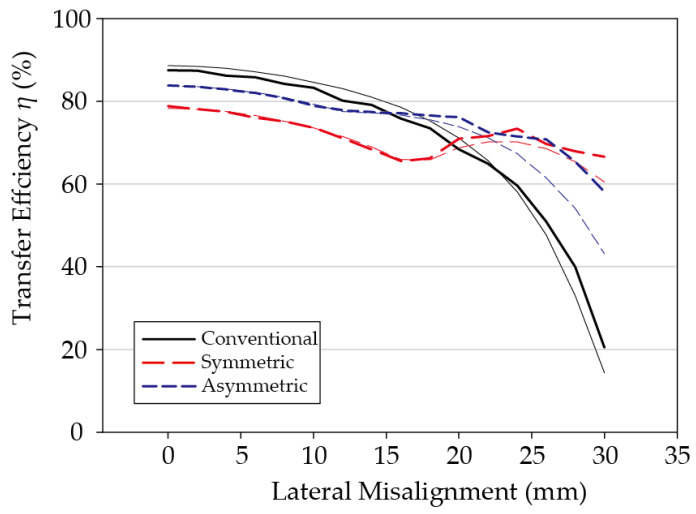
Comparison of the symmetric and asymmetric multilayer coil systems with the conventional system: simulated (thin) and measured (thick) results.

When multilayer technology is applied, a volume increase is inevitable. Therefore, realistically, it may be difficult to apply it in small systems, such as mobile devices, where it may be more practical to use an asymmetric structure where the multilayer coil technology is applied to the transmitter, but not to the receiver. To verify the effectiveness of the multilayer coil technology in the asymmetric system, we repeated the experiment by removing the assistant coil from the Rx coil stack. These results are shown in Figure 7. For comparison, the simulation test results are shown together, and the results for the conventional coil pair, to which the multilayer coil technology was not applied at all, are also shown. Similarly, in all cases, the measurement was performed by generating misalignments in the +*x* direction while maintaining the 7 mm for the minimum vertical distance between the Tx and Rx coils.

Because the assistant coil has been removed from the Rx coil stack in the asymmetric system, the distance between the primary coil pair Tx*_A_* and Rx*_A_* is reduced to maintain the vertical distance between the Tx and Rx coils at 7 mm. In other words, the distance between the primary coil pair decreases from *D_z_* = 11.5 mm to 9.23 mm in the symmetric system. Therefore, the efficiency between the primary coil pair increases, and because the assistant coil has been removed, the effect of the Eddy currents decreases, which leads to an additional increase in efficiency. Hence, in the experimental results, the maximum transfer efficiency of the asymmetric system in the perfectly aligned state *D_x_* = 0 mm is 83.8%, which is 4.9%P higher than that of the symmetric system. This indicates that the difference from the conventional system without the application of the multilayer coil technology is reduced by 3.7%P in the perfectly aligned state.

### 3.3. Summary of Experimental Results

Even if the number of assistant coils is reduced, the efficiency compensation effect with respect to misalignment remains considerable. For example, in the case of a 30 mm misalignment, i.e., a very large misalignment state of 67% compared to the maximum size of the coil, the transfer efficiency of the asymmetric system is 58.1%, which is much higher than the 20.8% transfer efficiency of the conventional coil system in the same misalignment state. Furthermore, it is comparable to the 66.6% efficiency of the symmetric system. In other words, the experiments show that efficiency compensation can be achieved even in the asymmetric system, in which the multilayer coil is applied to only one side. Table 4 comparatively summarizes the measurement results for the three coil systems.

Compared with [15], the coils in this work show relatively low efficiency. This is partly due to the smaller coil size and much lower frequency of operation. Further, the thicker assistant coils induce more loss, which is evidenced by the lower efficiency when the Tx and Rx coils are perfectly aligned. Nevertheless, the stacked coil technique in this work shows improved performance over [15] in terms of compensation of lateral misalignment. In [15], the efficiency maintains above 80% of the efficiency in the perfectly-aligned state up to 72% misalignment. This is an improvement of 22.2% over the conventional system. In this work, relative efficiency of 80% is maintained up to 67% misalignment. This is a 67.5% increase from the conventional system, which is substantially higher than the planar coils with much larger coils at 6.78 MHz [15]. It is even higher than that in [5], where the 80% relative efficiency is maintained up to approximately 55% misalignment. This verifies the effectiveness of the stacked coil technique in compensating for the lateral misalignment in a more practical system for circular coils consisting of non-planar litz wires in the kHz range.

## 4. Discussion

In this study, we applied a multilayer coil technology in the 100 kHz band to circular coils made of Litz wire and verified its effect on efficiency compensation according to the misalignment and efficiency of the aligned state. In the perfectly aligned state, an 8.6%P decrease in efficiency was observed compared to the conventional coil because the impact of the Eddy currents, occurring due to the assistant coil, is much larger. However, the effect of compensating for the decrease in efficiency due to misalignment is still excellent. Even if a misalignment of 30 mm, i.e., a misalignment corresponding to 67% of the coil size occurs, high transfer efficiency is shown—46.1%P higher than the 20.5% of the conventional coil. Furthermore, even in the asymmetric system in which the multilayer coil technology is applied to the Tx coil only, the efficiency increases to 58.1% in the state of 30 mm misalignment. In this work, the effect of the multilayer coil technology is demonstrated at 145 kHz. Although it is not shown here, simulated results reveal that the overall efficiency decreases slightly at 100 kHz due to the decrease in the *Q*-factor of the coils. However, the effect of compensating for the misalignment is similar enough that it is reasonable to expect the performance of multilayer technique to be as excellent in 100 kHz.

## Figures and Tables

**Table 1:** Final design parameters.

Parameter	Primary Coil	Assistant Coil
Outer diameter (*R_o_*)	45.3 mm	-
Inner diameter (*R_i_*)	33.2 mm	-
Width (*W*)	-	22.4 mm
Length (*L*)	-	45.3 mm
# of turns (*N*)	5	3

**Table 2:** Calculated coupling coefficients for various misalignment states.

*D_x_* (mm)	0		10		22	
Coil Pair	Tx*_A_* − Rx*_A_*	Tx*_B_*_2_ − Rx*_B_*_1_	Tx*_A_* − Rx*_A_*	Tx*_B_*_2_ − Rx*_B_*_1_	Tx*_A_* − Rx*_A_*	Tx*_B_*_2_ − Rx*_B_*_1_
Maxwell	0.262	−0.024	0.209	0.080	0.094	0.26
HFSS	0.266	−0.023	0.211	0.084	0.099	0.27

**Table 3:** Simulated and measured electrical parameters of designed coils.

	Simulated Results		Measured Results	
	Primary Coil	Assistant Coil	Primary Coil	Assistant Coil
Resistance (mΩ)	44	21	35	18
Inductance (μH)	1.48	0.48	1.76	0.59
Q-factor	30.6	20.7	45.8	31.7

**Table 4:** Summary of the measured performance.

	Conventional	Symmetric	Asymmetric
*η* @ *D_x_* = 0 mm	87.5%	78.9%	83.8%
*η* @ *D_x_* = 30 mm	20.5%	66.6%	58.1%
*η* ≥ 60%	≤24 mm	≤30 mm	≤29 mm
Switching point	-	17 mm	13 mm
